# A new species of *Phoebe* (Lauraceae) from south-western China

**DOI:** 10.3897/phytokeys.140.47664

**Published:** 2020-03-04

**Authors:** Bing Liu, Wei-Yin Jin, Li-Na Zhao, Yong Yang

**Affiliations:** 1 State Key Laboratory of Systematic and Evolutionary Botany, Institute of Botany, Chinese Academy of Sciences, Beijing 100093, China Institute of Botany, Chinese Academy of Sciences Beijing China; 2 Sino-Africa Joint Research Center, Chinese Academy of Sciences, Wuhan 430074, China Sino-Africa Joint Research Center, Chinese Academy of Sciences Wuhan China; 3 University of Chinese Academy of Sciences, Beijing, China University of Chinese Academy of Sciences Beijing China

**Keywords:** China, Lauraceae, *
Phoebe
*, taxonomy, Yunnan

## Abstract

Here *Phoebe hekouensis* Bing Liu, W.Y. Jin, L.N. Zhao & Y. Yang from south-eastern Yunnan Province of China is described as new to science. This species is morphologically similar to *P. megacalyx* H.W. Li in the twigs being robust and brownish tomentose, the ovary densely pubescent and the tepals longer than 1 cm, but differs from the latter species by the leaves being broader, up to 18 cm (vs. 4.5–11.5 cm), the inflorescences shorter, ca. 10–15 cm long (vs. up to 23 cm), the ovary completely and densely pubescent (vs. pubescent only at the apical portion) and the stigma conspicuous (vs. inconspicuous). The new species also resembles *P. macrocarpa* C.Y. Wu, but differs from the latter by the tepals being longer, 9–13 mm long (vs. ca. 4 mm).

## Introduction

The genus *Phoebe* Nees of the Lauraceae contains about 100 currently recognised species and is widely distributed in tropical and subtropical Asia (van der Werff 2001, Wei and van der Werff 2009). Members of this genus are usually trees, with pinnately-veined leaves usually obovate to oblanceolate and slightly clustered at the tips of branches and trimerous bisexual flowers with nine 4-loculed fertile stamens and persistent tepals clasping the base of the fruit. Recent molecular systematic studies have suggested that this genus appears to be monophyletic (Rohwer et al. 2009, Li et al. 2011a, 2011b, Song et al. 2017).

Traditionally, the genus *Phoebe* is classified into two sections, based on the pubescence of the tepals and inflorescences: sect. Phoebe possessing glabrous or appressed puberulent tepals and inflorescences and sect. Caniflorae Meisn. having densely pubescent/tomentose tepals and inflorescences (Lee and We 1982). This classification, however, is not supported by molecular phylogenetic studies, sect. Caniflorae being paraphyletic because a few species of this section actually fall within the clade of sect. Phoebe to which the type species belongs (Li et al. 2011a, 2011b, Song et al. 2017). The genus is in need of reclassification based on further molecular study involving more extensive sampling and examination of morphological characters.

A few species of the sect. Caniflorae do comprise a robust clade, for example, *P. macrocarpa* C.Y. Wu, *P. megacalyx* H.W. Li, *P. glaucophylla* H.W. Li and *P. hungmaoensis* S. Lee (Song et al. 2017). These species usually have robust twigs with dense indumentum, long leaves up to 40 cm (except for *P. hungmaoensis* that has shorter leaves ca. 10–15 cm), paniculate inflorescences possessing a long peduncle and branched only in the distal portion, pubescent ovaries and large fruits. We collected a specimen of *Phoebe* in south-eastern Yunnan Province, China, in April 2014. Further phylogenetic studies based on nuclear ITS and chloroplast *psb*A-*trn*H suggests that this plant belongs to the clade of *P. megacalyx* and *P. macrocarpa* (Jin 2017), but it clearly differs from all known species of this clade. As a result, we here describe this species as new to science. For identification purposes, we also provide a key to all known members of this particular clade.

## Materials and methods

We conducted field investigations during 2010 and 2014. Photographs were taken in the field. Morphological observations and measurements of the new species were made, based on both living plants and dry specimens.

## Taxonomy

### Phoebe hekouensis

Taxon classificationPlantaeLauralesLauraceae

Bing Liu, W.Y. Jin, L.N. Zhao & Y. Yang sp. nov.

808C0202-6371-5AC3-ABD7-47833E189EE9

urn:lsid:ipni.org:names:77206950-1

Figs 1 , 2

#### Type.

China. Yunnan: Hekou Yao Minority Autonomous County, Nanxi Town, Hua-Yu-Dong, alt. ca. 140 m elev., 5 Apr 2014, *Bing Liu, Y. Yang, Q.W. Lin, L. Jiang & X.J. Li 1988* (Holotype: PE; Isotypes: PE).

#### Diagnosis.

This new species resembles *P. megacalyx* in having tomentose twigs and large tepals, but differs from the latter species by broader leaves (12–18 cm vs. 4.5–11.5 cm), shorter inflorescences (10–15 cm vs. up to 23 cm), densely pubescent ovary (vs. only pubescent at the apical portion) and the enlarged stigma (vs. inconspicuous); also similar to *P. macrocarpa* in that the twigs of both being robust and tomentose, but distinguished by the longer tepals ca. 9–13 mm (vs. ca. 4 mm).

**Figure 1.**
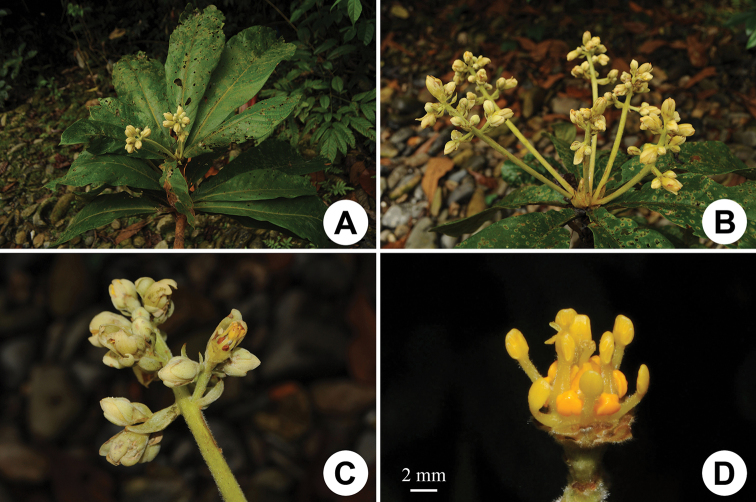
Morphology of *Phoebe hekouensis*. **A** Flowering branch, showing oblanceolate leaves and terminal inflorescences **B, C** inflorescences, these being panicles with long peduncles and branched only in the distal portion **D** flower with tepals removed, showing stamens, glands and staminodes.

**Figure 2.**
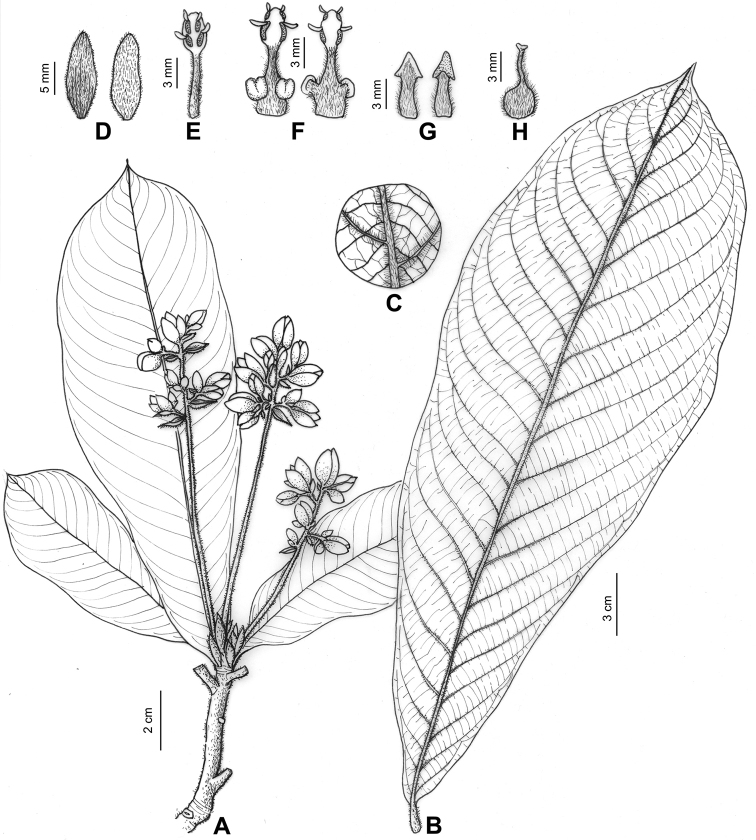
Illustration of *Phoebe hekouensis* to show morphological details. **A** Flowering branch **B** leaf, showing the oblanceolate shape and the ascending principal lateral veins **C** leaf portion magnified to show veinlet reticulations **D** tepals, depicting shape and pubescence of adaxial and abaxial side **E** fertile stamen of the first and second whorls **F** fertile stamens of the third whorl **G** staminodes, note sagittate head and pubescent stalk **H** pistil, showing pubescence.

#### Description.

Trees, ca. 12 m tall, bark greyish-brown. Branchlets robust, ca. 9 mm in diam., ridged, densely brown tomentose, possessing prominent dispersed leaf scars and clustered bud scale scars. Leaves alternate, usually clustered to somewhat verticillate at the apex of branches, coriaceous, oblanceolate, 25–45 × 12–18 cm, apex acuminate, base acute, upper surface glabrous, midrib impressed on the upper surface, principal lateral vein 18–30 pairs, immersed in the upper surface, both the midvein and the lateral veins prominently elevated on the lower surface, yellowish pubescent; petioles 1–2 cm long, brown tomentose. Inflorescences paniculate, 2–6 clustered at the apex of branches in between the clustered leaves; panicles robust, many-flowered at the apex, 10–15 cm long, densely yellow-brown tomentose, not branched in the lower half, usually few-branched in the distal portion and the flowers appearing to be clustered at the apex; peduncles 7.5–8.5 cm long, more than 2/3 of the total length, tomentose; bracts 2 cm long, tomentose. Flowers yellowish-white; subsessile. Bracts linear, brownish tomentose. Tepals in two whorls, subequal in length, elliptic, tepals of the outer whorl 9–13 mm long, ca. 6 mm broad, those of the inner whorl linear and narrower, ca. 4 mm broad, brownish tomentose on both sides. Fertile stamens 9, 4-loculed, the four locules arranged in trapezoid pattern; filaments 4–6 mm long, brownish pubescent, each filament of the third whorl bearing two yellow glands at the base; glands ovoid, stalked, stalks pubescent. Staminodes sagittate, ca. 4–6 mm long, possessing pubescent stalks. Ovary obovoid, brown tomentose; style straight and thread-like, ca. 3 mm long, pubescent, glabrescent toward distal end; stigma enlarged, disc-shaped. Flowers collected in April. Fruit not seen.

#### Distribution.

**Figure 3.**
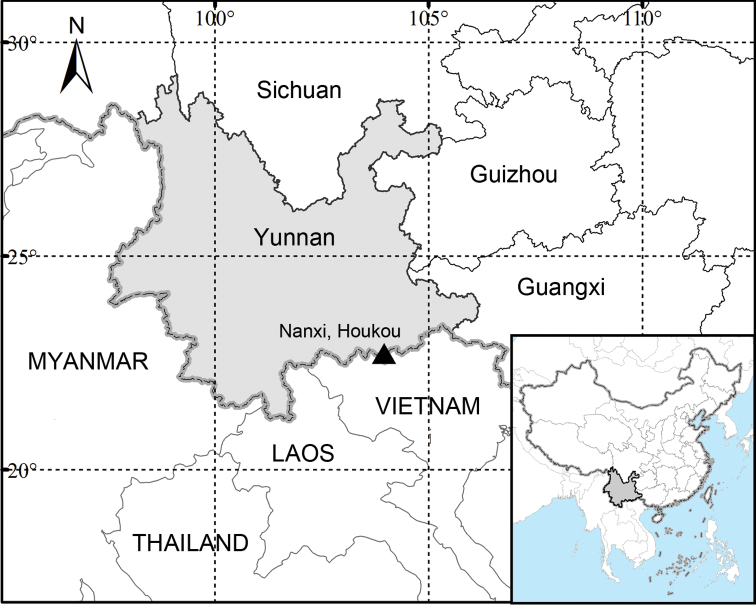
Distribution map showing the only known locality of *Phoebe hekouensis* (▲).

China. Yunnan, Hekou Yao Minority Autonomous County (Fig. 3).

#### Habitat.

In limestone ravines, near water.

#### Etymology.

The epithet “*hekouensis*” is after the type locality Hekou Yao Minority Autonomous County of Yunnan Province, south-western China.

#### Preliminary conservation status.

We have conducted field investigations in south-eastern Yunnan Province of China for ten years, but have found only one tree at the type locality, and no fruiting specimens were observed. It is uncertain if the species is endemic to China or is also distributed in adjoining Vietnam due to lack of field investigations in Vietnam. Based on IUCN Red List Categories and Criteria (IUCN 2012), we considered the new species as Critically Endangered (CR) in China. To conserve the species, we propose to take actions on reproduction of the tree in botanical gardens in the future.

### Key to the closely related species of *Phoebe* in the clade to which *P. hekouensis* belongs

**Table:** 

1	Tepals usually 10 mm or longer	**2**
–	Tepals shorter, ca. 4 mm	**3**
2	Leaves relatively narrow, 4.5–11.5 cm wide; inflorescences up to 23 cm long; stigma inconspicuous, punctiform	***P. megacalyx***
–	Leaves relatively broad, 12–18 cm wide; inflorescences 10–15 cm long; stigma conspicuous, disc-like	***P. hekouensis***
3	Fruits 3–4 cm long	***P. macrocarpa***
–	Fruits 1–2 cm long	**4**
4	Twigs usually glabrous; fruits ca. 1.8 cm long	***P. glaucophylla***
–	Twigs stout, pubescent; fruits shorter than 1.5 cm	**5**
5	Leaves relatively large, 12–23×5–9 cm long; pubescence brownish; fruits ovoid	***P. puwenensis* W.C. Cheng**
–	Leaves relatively small, 10–15×2–4.5 cm long; pubescence rusty; fruits ellipsoid	***P. hungmaoensis***

## Supplementary Material

XML Treatment for Phoebe hekouensis
